# Hydrocele is a benign pathology, an appearance disorder: no, this may be a common misconception

**DOI:** 10.3389/fruro.2026.1760748

**Published:** 2026-03-05

**Authors:** Ayhan Verit, Mert Verit, Fatma Ferda Verit

**Affiliations:** 1 Fatih Sultan Mehmet Hospital, Department of Urology, University of Health Sciences, Istanbul, Türkiye; 2 Hamidiye Medical Faculty, University of Health Sciences, Istanbul, Türkiye; 3 Haydarpaşa Hospital, Department of Gynecologic Oncology, University of Health Sciences, Istanbul, Türkiye

**Keywords:** hydrocele, male infertility, male sexual dysfunction, testicular cancer, thermo-stress

## Introduction

Hydrocele (Hc) is classically defined as a painless scrotal condition characterized by excessive fluid accumulation between the visceral and parietal layers of the processus vaginalis, and the etiology of idiopathic Hc remains largely unclear (1). Hc is generally regarded as a benign scrotal disorder. Although it is most commonly discussed in men, a homologous entity in women is the canal of Nuck cyst; Hc may cause physical discomfort and body-image concerns in men and may be congenital or acquired (2). Canal of Nuck cysts are rarely diagnosed in adult women and typically present as a fluctuating inguinal mass mimicking hernia, with or without pain, and are not usually associated with body-image concerns (3). Several classification systems describe different subtypes; however, these are beyond the scope of the present discussion. Hydrocelectomy is commonly recommended only when the condition becomes symptomatic or is complicated by an infection, hematocele, or acute scrotum (4). Efforts to evaluate the effects of Hc on intrascrotal temperature and fertility date back to the mid-20th century; early studies concluded that no meaningful association existed. Notably, however, almost no further research has been conducted since then (5, 6). In contrast, the hypothesis linking varicocele (Vc) to impaired spermatogenesis—primarily via increased intrascrotal temperature due to venous stasis in the pampiniform plexus—has remained current and has continued to expand (7). It appears paradoxical that one paratesticular fluid-related condition (blood stasis in Vc) is widely considered to impair fertility through heat stress, whereas another (Hc fluid) is often assumed to have no comparable effect. Indeed, Hc may plausibly exert a more pronounced adverse influence on fertility than Vc. Some authors have proposed that Hc may contribute to infertility by maintaining persistent heat stress through the insulating effect of the contained fluid mass (2). Basic thermodynamic principles support this premise, as the amount of heat transferred is directly proportional to liquid mass (8). As several heat-regulatory mechanisms maintain testicular temperature below core body temperature, these mechanisms may become insufficient when Hc fluid creates persistent pressure and a larger heat-retaining mass around the testis. Both the duration and magnitude of such thermal stress could therefore adversely affect spermatogenesis. In this study, we revisit this historically underestimated topic in light of the current literature, challenge the traditional approach, and argue—based on our interpretation—that surgical correction may warrant broader consideration.

## Main text

**Figure 1:**
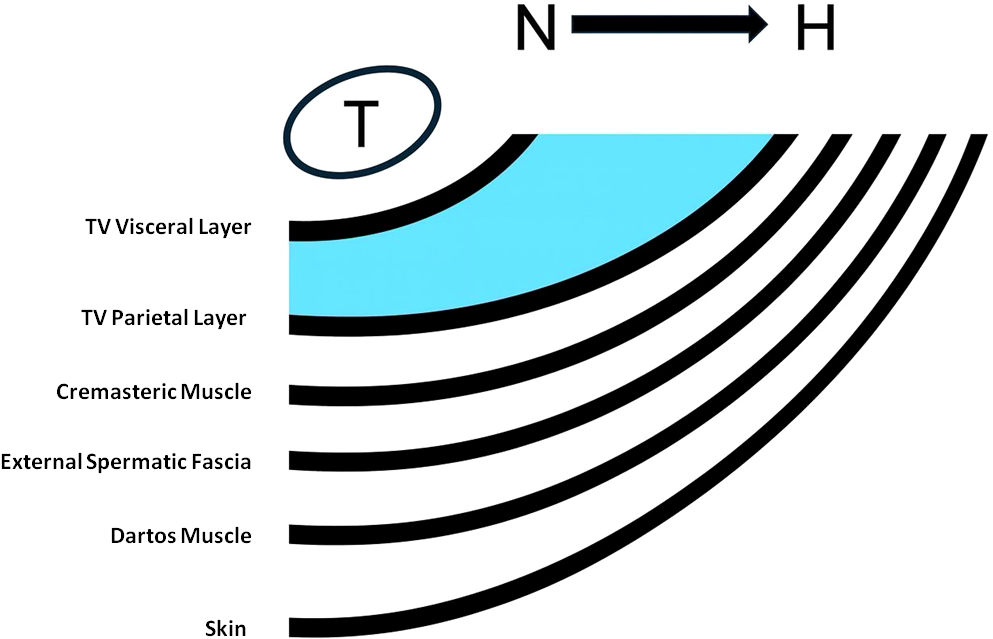
A schematic diagram of scrotal layers and hydrocele (H) fluid is shown, progressing from normal (N) to pathologic, from left to right. Tunika vaginalis (TV) and testes (T).

The cremaster muscle (Cm) is central to the cremasteric reflex and thereby to testicular thermoregulation, and it is innervated by the genital branch of the genitofemoral nerve. Cm fibers include both striated and smooth muscle components and may be physiologically significant due to multiple motor end plates. The genitofemoral nerve arises from the T12 and L2 spinal roots and provides sensory innervations to the upper thigh, scrotum, and scrotal contents; motor innervations to the Cm; and sympathetic innervations to the Dartos muscle (Dm) (9). Dm also contributes to scrotal reflexes and is similarly innervated via the genital branch of the genitofemoral nerve. Anatomical cross-sectional studies identify the Dm as a prominent scrotal layer consisting of interwoven smooth-muscle bundles arranged as an irregular network, with wide spaces between decussating bundles; this architecture facilitates a reduction in cutaneous surface area during contraction. Cremasteric and dartos reflexes are often conflated clinically; nonetheless, both are considered to contribute to scrotal thermoregulation and may therefore be relevant to the pathogenesis of male infertility (10). The principal role of the Cm is thought to be regulation of paratesticular temperature, maintaining an optimal testicular temperature of 34°C–35°C for spermatogenesis by contracting and relaxing. Through this mechanism, the testis is moved closer to, or farther from, the abdomen as a constant heat source (36°C–37°C), in accordance with environmental temperature variability (11). It is therefore reasonable to hypothesize that this heat-regulatory mechanism may become dysfunctional in the presence of Hc, due to the combined effects of fluid pressure and increased mass around the testis, with compression of muscle fibers within the scrotal compartment (**Figure 1**). Furthermore, basic physical principles suggest that Hc severity may influence the likelihood of spermatogenic impairment by disrupting optimal intratesticular temperature as a function of both heat magnitude and exposure duration. Evidence consistently indicates that thermal stress adversely affects testicular function, particularly spermatogenesis, in keeping with the evolutionary observation that the testes of most land mammals are located outside the body cavity to maintain a scrotal temperature 2°C–4°C lower than core body temperature (12). Even the debated topic of global warming has been associated with infertility through the detrimental effects of environmental heat exposure on the reproductive capacity of animal models, although these findings may not be directly adaptable to humans (12). Nonetheless, it has been shown that germ–cell maturation in low-temperature environments is associated with temperature-sensing properties of spermatozoa, including temperature-driven migration (thermo-taxis) (13).

From a thermodynamic standpoint, larger masses retain heat more effectively than smaller ones under identical environmental conditions. The volume of Hc fluid typically exceeds the volume of blood within dilated scrotal veins; consequently, Hc may exert a stronger thermodynamic effect on testicular temperature than Vc, with potential implications for fertility, although this hypothesis requires validation (14). In addition, high-grade Vc has been associated with Leydig cell apoptosis and reduced testosterone levels, which further supports the broader concept that chronic accumulation of scrotal fluid in Hc may affect male sexual health and endocrine function and raises the question of whether indications for Hc correction should be reconsidered in this context (15, 16). Moreover, an enlarged scrotum due to Hc is more likely to be in sustained contact with adjacent surfaces, particularly the thighs, which may facilitate equilibration of scrotal and core body temperatures. By contrast, Vc-associated infertility is not attributed solely to increased intrascrotal temperature; venous metabolic toxicity has also been counted in the etiology (17). Nevertheless, (i) recent proteomic studies assessing heat shock proteins in seminal plasma among men with Vc, and (ii) observations that increased heat is among the proposed contributors to infertility in obese patients, have strengthened thermo-stress-based hypotheses relevant not only to Vc but also to the present discussion (18, 19).

Hanley (1955) and Krahn (1963) previously examined the relationship between fertility and Hc (5, 6). Although these studies were pioneering, they do not meet contemporary methodological standards, given small sample sizes, limited statistical approaches, absent or unclear inclusion and exclusion criteria, and inadequate control of confounding variables. Krahn and colleagues reported intrascrotal temperature differences between the affected side and the contralateral control side that were not statistically significant; nevertheless, a measurable heat rise beneath the Hc was noted (6). In the current literature, the incidence of clinically apparent Hc has been reported in the general population as 0.1%, rising to approximately 3% among infertile men (20). Even these descriptive data suggest a potentially meaningful association and support the possibility that Hc severity could be linked to progressive impairment of spermatogenesis. Vc has also been reported to adversely affect testosterone levels through reduced Leydig cell function (21). At present, we do not assume an established detrimental effect of Hc on male sexual health via Leydig cell insufficiency due to the lack of direct evidence; however, an effect remains scientifically plausible and merits investigation. This gap in the literature may represent an additional rationale for considering Hc correction within a broader set of indications relevant to the aims of the present study. Varicocelectomy is among the most frequently performed operations for possible improvement of male infertility, although the level of certainty varies, and as a complication, postoperative Hc rates have been reported with wide variability (0%–29%) (22). In our view, when Hc occurs in this context, correction should be strongly considered in this infertile group. Additionally, we propose that chronic thermal stress within the scrotal environment, potentially including Hc might be relevant to oncologic risk in a manner analogous to the risk observed in intra-abdominal testes (23). We hypothesize that similar to undescended testes, Hc may increase scrotal testicular temperature toward core body levels, and thus, Hc could warrant consideration for surgical correction due to potentially shared thermogenic risks; however, this remains to be investigated. To our knowledge, direct evidence linking Hc or Vc to testicular cancer is lacking, and even the association with infertility remains insufficiently defined; nevertheless, the potential interrelationships among these factors merit further study.

In a controlled pathological study, testes with Hc demonstrated histological atrophy, with macroscopic flattening and microscopic partial or complete arrest of spermatogenesis (24). Beyond excess heat, chronic pressure on the testes due to Hc may increase intratesticular inflammatory responses, and chronic inflammation is widely recognized as a predisposing factor in basic tumor pathogenesis, influencing multiple steps of tumorigenesis (25). Dagur et al., in their study conceptually aligned with the present work, discussed pathophysiological mechanisms related to heat retention by scrotal fluid in the context of Hc and infertility, and asserted—without accompanying data—that “Hydrocele has a direct link to male infertility” (2). The severity of Hc may contribute to impaired spermatogenesis through persistent heat retention, although this remains hypothetical. They also reviewed prior pathological observations, including testicular atrophy, flattening, spermatogenic arrest attributed to impaired intrascrotal circulation due to mass effect, and structural changes such as thickening of scrotal layers including the basement membrane, tunica albuginea, and tunica vaginalis (**Figure 1**). However, testosterone-related dysfunctions beyond fertility, as well as oncologic risks, as discussed in the present study, were not addressed in their analysis. In a short letter with a limited cohort and without a control group, Osegbe reported reduced spermatogenic activity in men with Hc; however, no substantive subsequent data have appeared in the literature on this topic (26). Akpo, in a case series of giant Hcs (4–6 L), discussed Hc and infertility and reported an infertility rate of approximately 10% based on lifetime live birth outcomes (27). We consider this conclusion likely to be an underestimation given the limited data. Conversely, Politoff et al. suggested that corrected Hc in pediatric patients did not adversely affect fertility later in life (28). Overall, these scattered and limited statements do not provide a scientifically robust explanation. It should also be emphasized that fertility research ultimately targets “live birth,” an outcome influenced by multifactorial determinants, and therefore, inherently difficult to attribute to a single exposure. We were therefore surprised that Hc continues to be widely regarded in urological literature as a simple and harmless condition, despite the lack of high-quality evidence. Furthermore, fundamental physical principles and established concepts in cancer pathophysiology raise the possibility that Hc may have detrimental effects on male sexual health and potentially an oncologic risk to the testis. The oncologic concept of “body climate,” incorporating heat stress as a component of cancer pathophysiology, has been discussed beyond testicular disease, and prior authors have similarly highlighted the underestimation of this link (29).

In contemporary practice, Hc and Vc are often considered common and relatively mild entities that do not require correction, reflecting the fact that all surgical procedures carry risks of complications. Reported complications of Hc surgery include surgical site infection (rarely severe, including Fournier’s gangrene), postoperative intrascrotal hematoma sometimes requiring reoperation, and wound-healing problems (30). Moreover, uncertainty regarding the ultimate clinical benefit of intervention can influence decision-making.

By empirical similarity, large ovarian cysts (≥10 cm)—particularly those with an inflammatory microenvironment such as ovarian endometriomas (chocolate cysts) —may impair ovarian reserve through chronic mechanical compression of the ovarian cortex and sustained local inflammation rather than thermo-stress. It is noteworthy, with respect to the aim of this study, that chronic pelvic inflammation is implicated in epithelial ovarian carcinogenesis. While urological literature recommends surgical correction of Hc only when symptomatic, complicated, or when underlying pathology cannot be excluded, large ovarian cysts undergo surgery more often (31). This clear recommendation for the analogous organ in the opposite sex also encouraged us to suggest correction surgery for Hc, which may be considered less invasive than its female counterpart. Nevertheless, as with the aforementioned pathology in female analogues, Nuck cysts cannot be related to oncologic and fertility risks due to their inguinal location (3).

## Conclusion

To conclude, Hc should not be considered merely an “innocent” disorder limited to body-image concerns. The present study—pending further investigation—challenges the traditional approach by proposing that surgical correction may have a broader role. We suggest that Hc may be associated with increased risks of infertility, male sexual dysfunction, and testicular cancer in a manner proportional to the fluid volume, and that this hypothesis warrants further investigation. At a minimum, the inference should not be dismissed prematurely, and research on this topic should not be abandoned.
